# MiR-142-5p/FAM134B Axis Manipulates ER-Phagy to Control PRRSV Replication

**DOI:** 10.3389/fimmu.2022.842077

**Published:** 2022-06-20

**Authors:** Kaifeng Guan, Qiuju Su, Kailin Kuang, Xiangge Meng, Xiang Zhou, Bang Liu

**Affiliations:** ^1^ Key Laboratory of Agricultural Animal Genetics, Breeding, and Reproduction of Ministry of Education & Key Laboratory of Swine Genetics and Breeding of Ministry of Agriculture, Huazhong Agricultural University, Wuhan, China; ^2^ The Engineering Technology Research Center of Hubei Province Local Pig Breed Improvement, Huazhong Agricultural University, Wuhan, China; ^3^ The Cooperative Innovation Center for Sustainable Pig Production, Huazhong Agricultural University, Wuhan, China

**Keywords:** PRRSV, miR-142-5p, FAM134B, ER-phagy, Type I interferon

## Abstract

Porcine reproductive and respiratory syndrome virus (PRRSV) can replicate its RNA genome in endoplasmic reticulum (ER) and utilize ER to facilitate its assembly and maturation. To maintain ER homeostasis, host cells initiate reticulophagy (known as ER-phagy) to effectively digest the stressed ER. In this study, we found that PRRSV infection subverted ER-phagy by downregulating ER-phagy receptor FAM134B. PRRSV-induced miR-142-5p directly targeted FAM134B and significantly promoted PRRSV replication. Meanwhile, siRNA-mediated depletion of FAM134B protein and overexpression of FAM134B mutant protein significantly disrupted ER-phagy and facilitated PRRSV replication. Furthermore, our results showed that FAM134B-mediated ER-phagy activated type I interferon signaling to inhibit PRRSV replication. Overall, this study reveals the important role of ER-phagy in PRRSV replication in a FAM134B-dependent manner. Our findings provide an insight into the pathogenesis of PRRSV and offer a theoretical basis for further development of antiviral therapeutic targets.

## Introduction

Porcine reproductive and respiratory syndrome (PRRS), also known as “blue ear” disease, is one of the severe infectious diseases that causes reproductive failure in pregnant sows and respiratory symptoms in pigs of all ages. PRRS has devastated pig industries for many years and caused enormous economic losses (1, 2). PRRS is caused by porcine reproductive and respiratory syndrome virus (PRRSV), a positive-stranded enveloped RNA virus, and PRRSV belongs to the order Nidovirales and the family Arteriviridae (3). PRRSV genome with a length of approximately 15 kb contains nine open reading frames (ORFs), of which ORF1a and ORF1b encode viral nonstructural proteins (NSPs), and ORF2-ORF7 encode viral structural proteins (4, 5). PRRSV mainly infects macrophages and monocytes, resulting in the subversion of innate and adaptive immune responses of pigs. PRRSV infection causes a series of physiological and structural changes in cells including membrane remodeling, which constitutes a microenvironment to facilitate PRRSV replication and assembly (6, 7).

The endoplasmic reticulum (ER) is membrane-enclosed tubules and sacs, and it plays vital roles in numerous biological processes (8). ER is closely associated with PRRSV replication and virion assembly. PRRSV originated from the vesicles of ER utilizes ER to facilitate its assembly and maturation through the secretory pathways (7). During PRRSV infection, the accumulated viral proteins, viral genomic RNA, and misfolded proteins cause ER stress and induce unfolded protein response (UPR) in the infected cells. UPR induced by PRRSV promotes viral RNA synthesis by hijacking activating transcription factor 4 (ATF4) into cytoplasmic replication complexes (9). Therefore, effective clearance of the stressed ER leads to degradation of unfolded protein through the ER-associated degradation (ERAD) and/or autophagy pathways, thus controlling ER quality, eventually limiting virus replication (10–15).

Autophagy, as a conserved cellular recycling mechanism, degrades unnecessary dysfunctional components and releases resources to maintain intracellular homeostasis (16). Autophagy is divided into non-selective autophagy and selective autophagy based on the contents of degradation. Selective autophagy degrades damaged organelles and misfolded proteins through specific receptors (17). These receptors can specifically recognize ER and deliver ER fragments into autophagosomes to mediate ER-phagy (16). Currently, six mammalian ER-phagy receptors including FAM134B (18), RTN3 (19), ATL3 (20), SEC62 (21), CCPG1 (22), and TEX264 (23) have been reported to mediate ER-phagy under different physiological or pathological conditions. Among these ER-phagy receptors, FAM134B is the first identified receptor, and it regulates the shape and size of the ER (8). FAM134B contains reticulon-homology domain (RHD) and LC3-interacting region (LIR) domain. The RHD of FAM134B is responsible for sensing and inducing ER membrane curvature, while LIR domain is responsible for interaction with LC3/GABARAP proteins to form autophagosomes (18, 24). FAM134B has been reported to participate in multiple biological processes, including virus replication (25), procollagen quality control (26), neuropathy (27), and preadipocyte differentiation (28). FAM134B is a limiting factor for virus infection and replication. FAM134B-mediated ER-phagy limits the replication of Ebola virus (EBOV), dengue virus (DENV), Zika virus (ZIKA), and West Nile virus (WNV) (14, 29). Moreover, viruses can disrupt FAM134B-dependent ER-phagy pathway, thereby hijacking ER and facilitating its replication. NS3 protease of flavivirus subverts ER-phagy by specifically and directly cleaving FAM134B protein (14). Flavivirus inhibits FAM134B-dependent ER-phagy *via* bactericidal/permeability-increasing protein (BPI) fold-containing family B, member 3 (BPIFB3), thereby facilitating viral replication (15). ER membrane remodeling and ER stress have been observed in PRRSV-infected cells (30). However, whether ER-phagy plays a role in ER membrane rearrangement and PRRSV replication remains to be further investigated. In this study, we found that PRRSV infection attenuated the expression of FAM134B protein, thus inhibiting ER-phagy, ultimately facilitating PRRSV replication, and that PRRSV inhibited FAM134B expression by upregulating miR-142-5p. Re-induction of FAM134B could effectively restrict PRRSV replication and assembly. Our data indicate that ER-phagy constitutes a potent antiviral pathway, and that miR-142-5p/FAM134B axis could be a potential target controlling PRRSV pathogenesis.

## Materials and Methods

### Animals, Cells, and Viruses

PK15^CD163^ (obtained from Prof. En-ming Zhou from Northwest A&F University), MARC-145, and PK15 cells were cultured at 5% CO_2_ and 37°C in Dulbecco’s modified Eagle’s medium (DMEM) (Gibco, South Logan, UT, USA) supplemented with 10% fetal bovine serum (FBS) (Gibco, South Logan, UT, USA). The porcine alveolar macrophages (PAMs) were cultured at 5% CO_2_ and 37°C in RPMI-1640 medium (Gibco, South Logan, UT, USA) supplemented with 10% FBS. PRRSV strain WUH3 (GenBank accession No.HM853673) was provided by Prof. Shaobo Xiao from Huazhong Agricultural University (31). Cells were infected with PRRSV for 1 h at 37°C, and then the culture medium was replaced with maintenance medium containing 2% FBS until cells were collected. To ensure the consistency of the expression levels of FAM134B protein in mock groups at different time points post infection, the DMEM medium containing 10% FBS was used. PRRSV infection experiments *in vivo* were performed according to the previously reported method (32). The PAMs were collected from PRRSV-infected pigs at 0 day post infection (dpi), 7 dpi, and 14 dpi, respectively.

### SiRNAs, Plasmids, and Transfections

SiRNAs targeting *FAM134B* (siFAM134B-1: CCACTGTTCGCAGAATCA, siFAM134B-2: GAAGGATACACTCCACAGA and siFAM134B-3: CCATCAAAGACCAGTTAGA) were synthesized by Ribobio (Guangzhou, China). The sequences of siRNA targeting *TLR3*, *RIG-I*, and *ATG7* were synthesized, as previously described (33, 34). pGL3-IFNβ (IFNβ-Luc) was provided by Dr. Likai Ji from Shanghai Jiao Tong University (35). Trelief™ SoSoo Cloning kit (Tsingke Biotechnology, Wuhan, China) was used to construct plasmids pcDNA3.1-FAM134B, eGFP-FAM134B, mCherry-LC3B, mCherry-SEC61B, psi-Check2-FAM134B-3’UTR (FAM134B-3’UTR), and CMV-mCherry-eGFP-RAMP4-IRES-Puro. In addition, site-mutagenesis plasmids FAM134B-LIR-MUT and FAM134B-3’UTR-MUT were synthesized by Tsingke Biotechnology. The miR-142-5p (CAUAAAGUAGAAAGCACUACU) mimics, inhibitor (AGUAGUGCUUUCUACUUUAUG), and negative control (NC, UUCUCCGAACGUGUCACGUTT) were synthesized by GenePharma (Shanghai, China). The 25 nM siRNA/mimics or 2ug plasmids were transfected into cells using Lipofectamine™ 2000 transfection reagent (Invitrogen™, Carlsbad, CA, USA) for subsequent experiments.

### Immunoblot

The total protein of cells was extracted using RIPA lysis buffer (Beyotime, Shanghai, China), PMSF (Beyotime, Shanghai, China), and phosphatase inhibitor Cocktail I (MedChemExpress, Shanghai, China). Total protein was subjected to electrophores using SDS-PAGE (90 V for 0.5 h and 120 V for an additional hour), and then was transferred to polyvinylidene difluoride (PVDF) membrane (Millipore, MA, USA). PVDF membranes were blocked with 5% non-fat dry milk for 2.5 h at room temperature, and subsequently incubated with specific primary antibodies overnight at 4°C. After being washed with TBST (0.01% Tween-20 in TBS) for three times, the membranes were then incubated with corresponding secondary antibodies for 2 h at room temperature. Immunoblot was performed using ImageQuant LAS4000 mini (GE Healthcare Life Sciences, Piscataway, NJ, USA). All antibodies are shown in **Table S1**.

### Microscopy

For immunostaining, cells were fixed with 15% paraformaldehyde for 15 min, and the membranes were permeabilized with 0.3% Triton X-100 for 10 min. Then, the cells were blocked with 5% bovine serum albumin (BSA) for 1 h at room temperature, and incubated with primary antibodies overnight at 4°C, followed by incubation with corresponding fluorescent secondary antibodies for 2 h at room temperature. The images were captured by confocal fluorescence microscopy (Zeiss LSM 800, Oberkochen, Germany) and analyzed by ZEN software (ZEISS, Oberkochen, Germany). The antibodies used are listed in **Table S1**. MARC-145 cells or PAMs were fixed with Glutaraldehyde (Solarbio, Beijing, China) and subsequently observed with a transmission electron microscope (HITACHI H-7000FA, Tokyo, Japan).

### Quantitative Real-Time PCR

Total RNA was extracted by RNAiso Plus (TAKARA, Tokyo, Japan), and reverse transcribed into cDNA using a RevertAid First Strand cDNA Synthesis kit (Thermo Fisher Scientific, Carlsbad, CA, USA). The cDNA was subjected to quantitative real-time PCR analysis using TB Green^®^ Premix Ex Taq™ (TAKARA, Tokyo, Japan) on a BioRad CFX384 system (Bio-Rad, Richmond, CA, USA). The details of the primers are listed in **Table S2**.

### Construction of Stable Cell Lines

Lentiviral eukaryotic expression vector CMV-mCherry-eGFP-RAMP4-IRES-Puro and helper vectors PMG2.G and PSPAX were co-transfected into the 293T cells at the mass ratio 3:2:1. At 48 h post co-transfection, the cell supernatant was collected and filtered with a 0.45 um filter to remove dead cells or cell debris. The collected cell supernatant was ultracentrifugated at 25000 g for 2 h. Then, 300 ul serum-free DMEM was added to obtain diluted lentivirus, and the diluted lentivirus was stored at -80°C until subsequent use. MARC-145 cells were infected with lentivirus and added with 1μg/ml polybrene to increase infection efficiency. The infected MARC-145 cells were sorted by flow cytometry to prepare single cell suspensions. The positive cells stably expressing GFP/mCherry were selected for subsequent experiments.

### 50% Tissue Culture Infectious Dose Assay

Briefly, MARC-145 cells were inoculated onto 96-well plates. When the confluence reached 80%, the MARC-145 cells were infected with 10-fold serially diluted PRRSV. The cells were incubated for 72 to 120 h, and then virus titers were measured. PRRSV titers were expressed as TCID_50_ per milliliter using the Reed-Muench method.

### Luciferase Reporter Assay

PK15 or PK15^CD163^ cells were inoculated onto 24-well plates. The cells were then subjected to dual-luciferase assay using a Dual-Luciferase Reporter Assay System (Promega, Wisconsin, USA) with the Enspire reader (PerkinElmer, Singapore) at 24 h post co-transfection with corresponding plasmids (500 ng) and pRL-TK (50ng). After co-transfection, PK15^CD163^ cells were infected with PRRSV for another 24 h for detecting the promoter activity of *IFN-β*.

### Flow Cytometry Analysis

After the cells were trypsinized and resuspended in complete medium without fixation, flow cytometry assay of cells was performed by ER autophagy tandem reporter (EATR). A total of 20000 cells were counted for each treatment. The acid gate was set according to previously-reported method (36). The data were then analyzed by eGFP fluorescence and mCherry fluorescence using FlowJo software.

### RNA Sequencing

PRRSV-infected PK15^CD163^ cells were transfected with FAM134B or control overexpression vector for 24 h. Then, total RNA was extracted for RNA-seq library construction. Subsequently, hisat2 software (37) was used to align the high-quality reads to the pig reference genome (ENSEMBL *Suscrofa11.1*). Finally, differential expression of genes in each sample was analyzed using edgeR software (38). The raw RNA-seq data were submitted to SRA database (NO. PRJNA773967).

### Statistical Analysis

All the data had at least three biological replicates, and they were presented as the mean ± SD. Kruskal Wallis test was performed for multiple comparisons, and Wilcoxon test was conducted for two comparisons. *P*-value < 0.05 and < 0.01 were considered as statistically significant and highly significant, respectively.

## Results

### PRRSV Infection Inhibits ER-phagy

**Figure 1:**
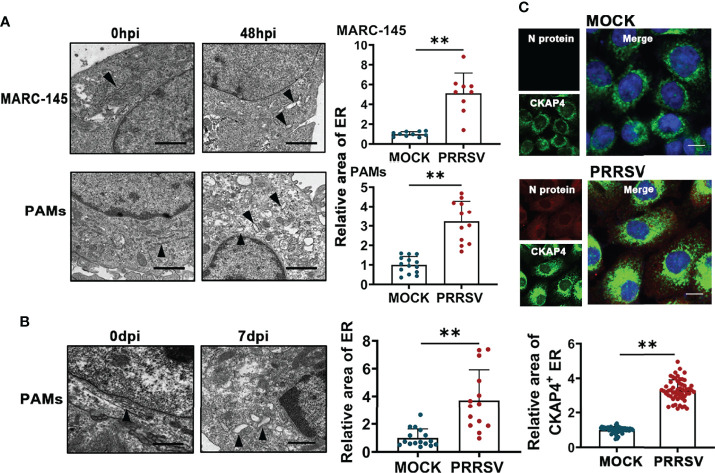
PRRSV infection results in ER expansion. **(A)** Representative TEM images of MARC-145 cells and PAMs infected with or without PRRSV (MOI=5) for 48 h and quantification of ER area (n>10). Scale bars: 2 μm. **P < 0.01, Wilcox test. **(B)** Representative TEM images of PAMs collected from pigs infected with or without PRRSV for 7 days *in vivo*. Black arrows indicate the subcellular localization of ER. The area of ER was quantified (n>10). Scale bars: 2 μm. **P < 0.01, Wilcox test. **(C)** Immunostaining of CKAP4 (Green) and PRRSV N (Red) in MARC-145 cells infected with or without PRRSV (MOI=2) for 48 h. Representative confocal images were shown, and the area of CKAP4^+^ ER was quantified (n>50). Scale bars: 20 μm. **P < 0.01, Wilcox test.

**Figure 2:**
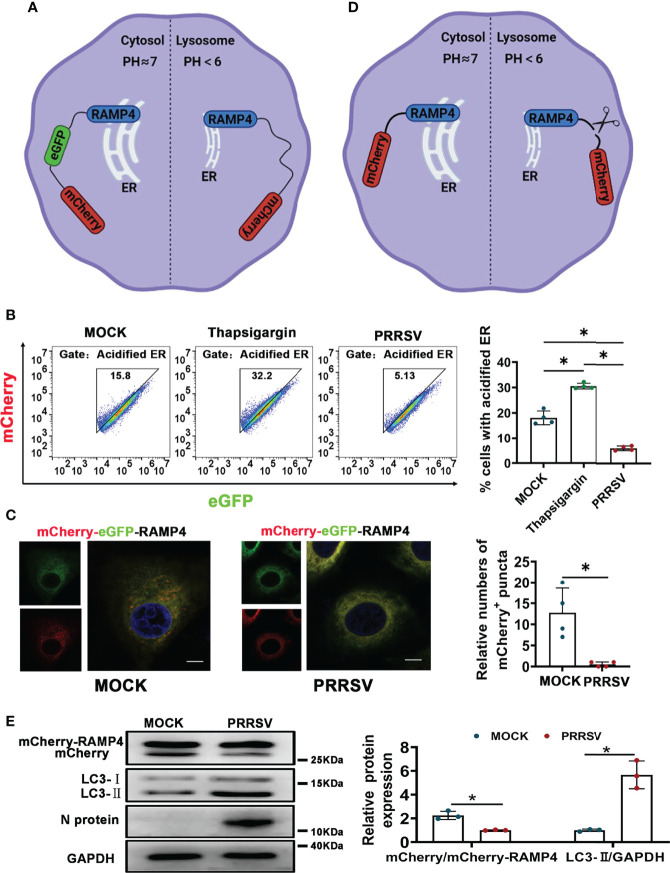
CCER and EATR assays of ER-phagy. **(A)** Schematic representation of EATR assay. The quenching of eGFP signaling in mCherry-eGFP-RAMP4 reporter indicated the occurrence of ER-phagy. **(B)** Flow cytometry analysis of EATR system infected with PRRSV (MOI=1) or treated with thapsigargin (0.5uM, positive control) for 48 h. *P < 0.05, Kruskal Wallis test, n=4. **(C)** Confocal images of EATR system infected with PRRSV (MOI=1) for 48 h. mCherry^+^ puncta were counted. Scale bars: 20 μm. *P < 0.05, Wilcox test, n=4. **(D)** Schematic representation of CCER assay. The cleavage of RAMP4-mCherry to form free mCherry suggested the occurrence of ER-phagy. **(E)** Expression of mCherry, mCherry-RAMP4, LC3-I, and LC3-II proteins in PK15^CD163^ cells transfected with mCherry-RAMP4 vector. The relative ratio of mCherry/mCherry-RAMP4 and that of LC3-II/GAPDH in PK15^CD163^ cells infected with PRRSV (MOI=1) for 36 h were calculated. GAPDH served as the control. *P < 0.05, Kruskal Wallis test, n=3.

PRRSV infection has been reported to change ER morphology and enlarge ER volume in the infected cells (30). Consistent with this report, through transmission electron microscopy, we observed ER expansion in the PRRSV-infected MARC-145 cells and the PAMs *in vitro* (**Figure 1A**) as well as in the PAMs from PRRSV-infected pigs *in vivo* (**Figure 1B**). Furthermore, we also observed that the staining of ER marker protein cytoskeleton-associated protein 4 (CKAP4) was enhanced in the PRRSV-infected MARC-145 cells (**Figure 1C**). Based on these observations, we hypothesized that ER-phagy, as the key step of ER-quality control, might affect ER morphology in the PRRSV-infected cells. To test this hypothesis, we constructed PRRSV-susceptible ER autophagy tandem reporter (EATR) system. PRRSV-susceptible EATR system was composed of MACR-145 cells stably expressing mCherry-eGFP-RAMP4 reporter (ER-colocalized RAMP4 fused with mCherry and eGFP) (**Figure 2A**). ER-phagy has been reported to deliver ER fragments and mCherry-eGFP-RAMP4 reporter into the acidic environment of the lysosomes, which deprives mCherry-eGFP-RAMP4 reporter of eGFP fluorescence but maintain its mCherry fluorescence (36). The flow cytometry assay showed that upon PRRSV infection, the number of cells with the acidified ER was significantly declined in the PRRSV-susceptible EATR system, but this number was significantly increased after treatment with thapsigargin (an ER-phagy inducer) (**Figure 2B**). In addition, the number of ER puncta with merely mCherry fluorescence was decreased in MARC-145 cells stably expressing mCherry-eGFP-RAMP4 reporter during PRRSV infection (**Figure 2C**). Furthermore, PRRSV-susceptible mCherry-Cleaved ER-phagy Reporter (CCER) system was also employed to investigate the effect of PRRSV infection on ER-phagy (**Figure 2D**). In CCER system, mCherry-RAMP4 will be cleaved into free mCherry in acidic environment of the lysosome (36). PRRSV-susceptible CCER system was constructed by transfecting mCherry-RAMP4 vector into MARC-145 cells. The immunoblot assay showed that the expression of free mCherry was significantly downregulated after PRRSV infection (**Figure 2E**). In addition, both PRRSV infection and BafA1 treatment promoted the accumulation of LC3-II, indicating that PRRSV could partially inhibit the fusion of autophagosomes and lysosomes (**Figure 2E** and **Figure S1**). These results suggested that PRRSV could subvert ER-phagy and change ER morphology in the infected cells.

### PRRSV Negatively Regulates FAM134B to Subvert ER-phagy

**Figure 3:**
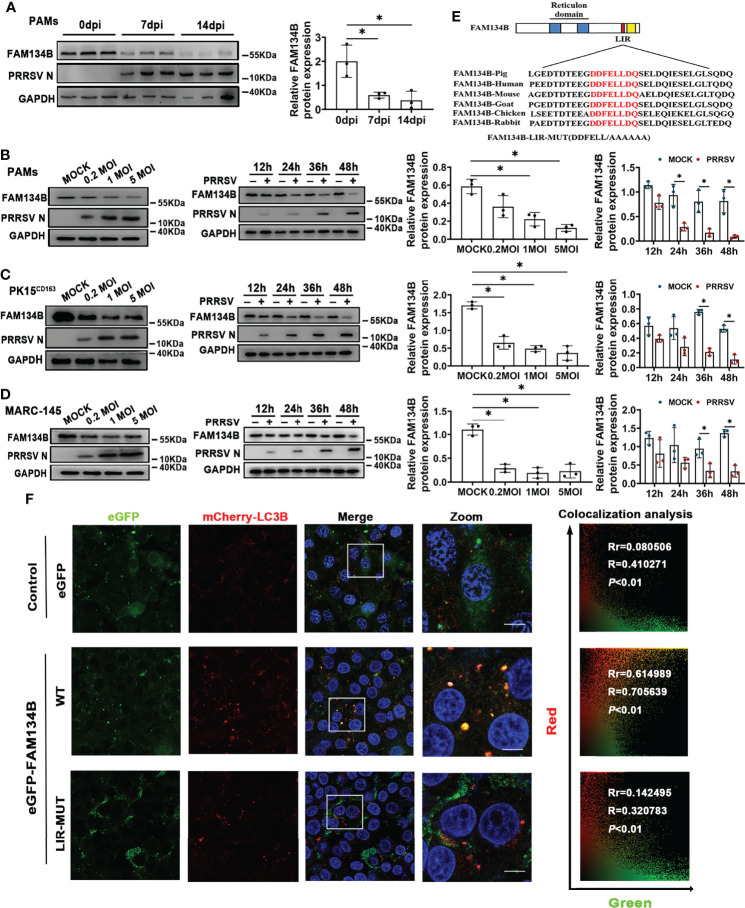
PRRSV infection downregulates FAM134B protein expression. **(A)** Expression of FAM134B and PRRSV N protein in PAMs.. PAMs were collected from pigs infected with PRRSV (TCID_50 =_ 10^6^) at 0 dpi, 7 dpi, and 14 dpi, respectively. *P < 0.05, Kruskal Wallis test, n=3. **(B–D)** Expression of FAM134B and PRRSV N protein in PAMs **(B)**, PK15^CD163^ cells **(C)**, and MARC-145 cells **(D)**. Cells were infected with different titers of PRRSV or at different time points, and these cells were subjected to immunoblotting assays. *P < 0.05, Kruskal Wallis test, n=3. **(E)** Schematic representation of FAM134B protein domains. The amino acid DDFELL in FAM134B protein’s LIR domain bound by LC3 protein was mutated into AAAAAA for constructing FAM134B-LIR-MUT vector. **(F)** Representative confocal images of PK15 cells co-transfected with mCherry-LC3B (red) and empty vector (green), or eGFP-FAM134B (green), or eGFP-FAM134B-LIR-MUT (green). Pearson’s correlation (Rr) and Overlap coefficient (R) were calculated. Scale bars: 20 μm. T distribution was used to find the *P*-value. GAPDH served as the control in immunoblotting assay.

Considering that FAM134B is the best-characterized ER-phagy receptor regulating ER turnover, we hypothesized that PRRSV might disrupt ER-phagy by suppressing FAM134B expression. To test this hypothesis, we investigated the expression of FAM134B protein in PAMs isolated from pigs at 7 or 14 days post PRRSV infection (dpi) *in vivo.* The results showed that the expression level of FAM134B protein was significantly decreased in the PRRSV-infected cells (**Figure 3A**). Consistent with the FAM134B expression pattern *in vivo*, PRRSV infection significantly downregulated the expression of this protein in PAMs (**Figure 3B**), PK15^CD163^ (**Figure 3C**), and MARC-145 cells (**Figure 3D**) *in vitro* at 36 hpi or 48 hpi. Additionally, PRRSV infection downregulated the expression of FAM134B protein in a dose-dependent manner (**Figures 3B–D**). Porcine FAM134B protein is homologous to human and mouse FAM134B containing typical RHD and LIR domain (**Figure 3E**). Porcine FAM134B was co-localized with ER markers CKAP4 and SEC61B (**Figure S2A**), and we observed starvation-induced degradation of FAM134B, CKAP4, and RTN4 (**Figure S2B**), indicating that FAM134B might be a major factor responsible for ER-phagy. The transient overexpression results of GFP-FAM134B and mCherry-LC3B in PK15 cells showed that mCherry and GFP puncta were co-localized. However, mutation of porcine FAM134B LIR motif abolished its colocalization with mCherry-LC3B in PK15 cells (**Figure 3F**), which was in line with the previous report in mouse model (14).

### PRRSV-Induced miR-142-5p Directly Targets FAM134B

**Figure 4:**
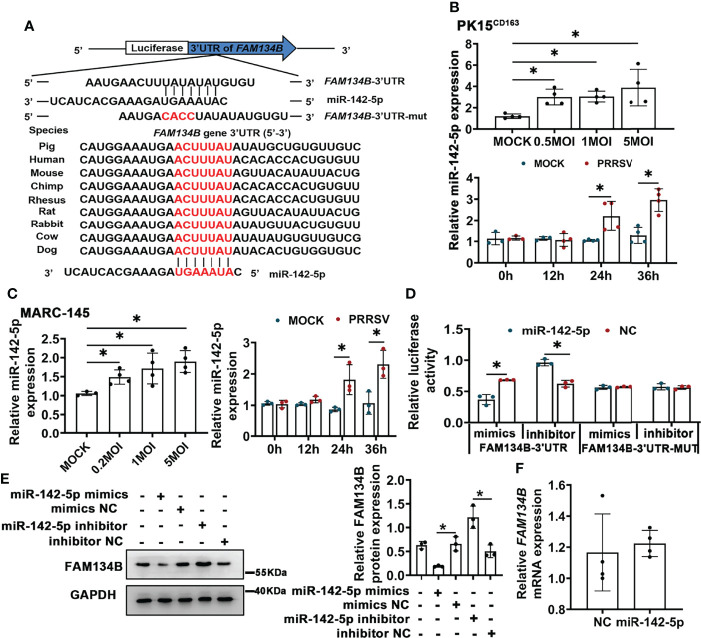
miR-142-5p targets 3’UTR of *FAM134B* and downregulates protein expression of FAM134B. **(A)** Binding site of miR-142-5p. FAM134B-3’UTR-MUT vector was constructed by mutating ACUU into CACC at the binding site in *FAM134B*-3’UTR. **(B, C)** Expression of miR-142-5p in PK15^CD163^ cells **(B)** and MARC-145 cells **(C).** Cells were infected with different titers of PRRSV or at different time points and were amplified by qPCR. *P < 0.05, Kruskal Wallis test, n=3 or 4. **(D)** Luciferase activity of *FAM134B*-3’UTR and *FAM134B*-3’UTR-MUT from PK15 cells transfected with miR-142-5p mimic or inhibitor. *P < 0.05, Kruskal Wallis test, n=3. **(E)** Immunoblotting assay of *FAM134B* in PK15 cells transfected with miR-142-5p mimics, mimics NC, miR-142-5p inhibitor, or inhibitor NC. *P < 0.05, Kruskal Wallis test, n=3. **(F)** mRNA expression of FAM134B from PK15 cells treated with or without miR-142-5p mimic for 24 h. n=4. GAPDH served as the control in immunoblotting assay.

PRRSV infection significantly downregulated FAM134B protein expression *in vivo* and *in vitro*. The miRNA-mediated gene regulation has been reported to be closely related to PRRSV pathogenesis (39). Our prediction by Targetscan software indicated that *FAM134B* gene was the best target of miR-142-5p, and that the binding sites of miR-142-5p to 3’ untranslated regions (UTR) of *FAM134B* were conserved across different species (**Figure 4A**). Our previous transcriptomic data have shown that the expression of miR-142-5p was significantly upregulated in PAMs in response to PRRSV infection (40). The expression of miR-142-5p was also upregulated in PRRSV-infected PK15^CD163^ cells and MARC-145 cells *in vitro* (**Figures 4B, C**). Dual-luciferase reporter assays confirmed that *FAM134B* gene was directly targeted by miR-142-5p. The luciferase activity of FAM134B-3’UTR reporter was significantly decreased when this reporter was co-transfected with miR-142-5p mimics into PK15 cells, while that was enhanced when this reporter was co-transfected with miR-142-5p inhibitor. The luciferase activity of FAM134B-3’UTR-MUT reporter remained unchanged when this mutant reporter was co-transfected with miR-142-5p mimics or inhibitor into PK15 cells (**Figure 4D**). The immunoblot assay showed that miR-142-5p mimics inhibited the expression of FAM134B in PK15 cells, whereas miR-142-5p inhibitor promoted it (**Figure 4E**). Furthermore, miR-142-5p had no effect on mRNA expression of *FAM134B* (**Figure 4F**).

### FAM134B-Mediated ER-phagy Inhibits PRRSV Replication

**Figure 5:**
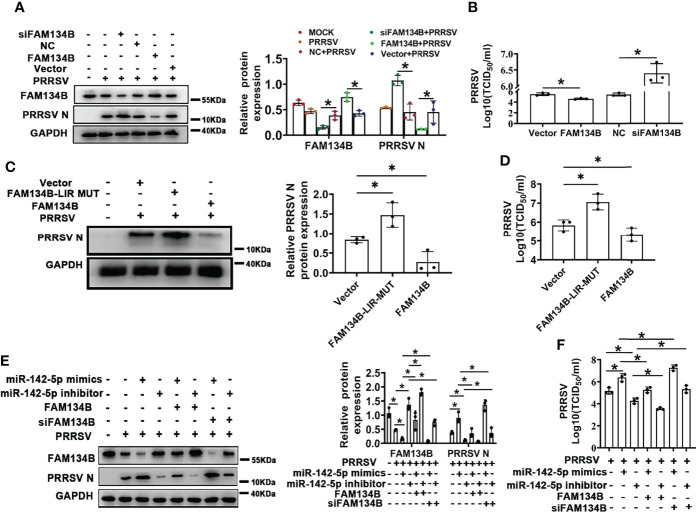
FAM134B inhibits PRRSV replication. **(A)** Expression of FAM134B and PRRSV N protein in PK15^CD163^ cells. FAM134B-overexpressed/FAM134B-depleted PK15^CD163^ cells were infected with PRRSV (MOI=1) for 48 h, and were subjected to immunoblotting assay. *P < 0.05, Kruskal Wallis test, n=3. **(B)** PRRSV titers in FAM134B-overexpressed or FAM134B-depleted PK15^CD163^ cells by TCID_50_ analysis. *P < 0.05, Kruskal Wallis test, n=3. **(C)** Expressions of PRRSV N protein in PK15^CD163^ cells. PK15^CD163^ cells were infected with PRRSV (MOI=1) for 48 h after transfection with eGFP-FAM134B or eGFP-FAM134B-LIR-MUT. *P < 0.05, Kruskal Wallis test, n=3. **(D)** PRRSV titers in PK15^CD163^ cells transfected with eGFP-FAM134B or eGFP-FAM134B-LIR-MUT by TCID_50_ analysis. *P < 0.05, Kruskal Wallis test, n=3. **(E)** Immunoblotting analysis of FAM134B and PRRSV N proteins in PK15^CD163^ cells in different groups. *P < 0.05, Kruskal Wallis test, n=3. **(F)** PRRSV titers in PK15^CD163^ cells in different groups by TCID_50_ analysis. GAPDH served as the control in immunoblotting assay. *P < 0.05, Kruskal Wallis test, n=3.

PRRSV replication and assembly have been reported to be closely associated with the ER (7). Considering this, we speculated that FAM134B-dependent ER-phagy might inhibit PRRSV replication. To test this speculation, FAM134B was transiently overexpressed in PRRSV-infected PK15^CD163^ cells to rescue PRRSV-induced downregulation of FAM134B. Additionally, the porcine *FAM134B* gene-targeting siRNA was transfected into PK15^CD163^ cells to enhance the downregulation of FAM134B. The results showed that the expressions of PRRSV N protein and *ORF7* as well as the titers of virus were significantly decreased in FAM134B-overexpressed PK15^CD163^ cells (**Figures 5A, B** and **Figure S3B**), whereas they were significantly increased in FAM134B-silenced PK15^CD163^ cells (**Figures 5A, B** and **Figures S3A–D**). In addition, overexpression of FAM134B-LIR-MUT protein failed to inhibit PRRSV replication, indicating that FAM134B could inhibit PRRSV replication by enhancing ER-phagy (**Figures 5C, D** and **Figures S3E, F**). Furthermore, the immunoblot assay showed that miR-142-5p promoted PRRSV replication (**Figure 5E**). Overexpression and silencing of FAM134B blunted or enhanced the effect of miR-142-5p on PRRSV replication, respectively (**Figure 5E**). Further, we tested the virulence of PRRSV produced by these cells, and found that TCID_50_ was negatively correlated with FAM134B, but positively with miR-142-5p (**Figures 5F**). In conclusion, all above data demonstrated that miR-142-5p promoted PRRSV replication by inhibiting FAM134B expression.

### FAM134B Activates Type I Interferon Signaling to Restrict PRRSV Infection

**Figure 6:**
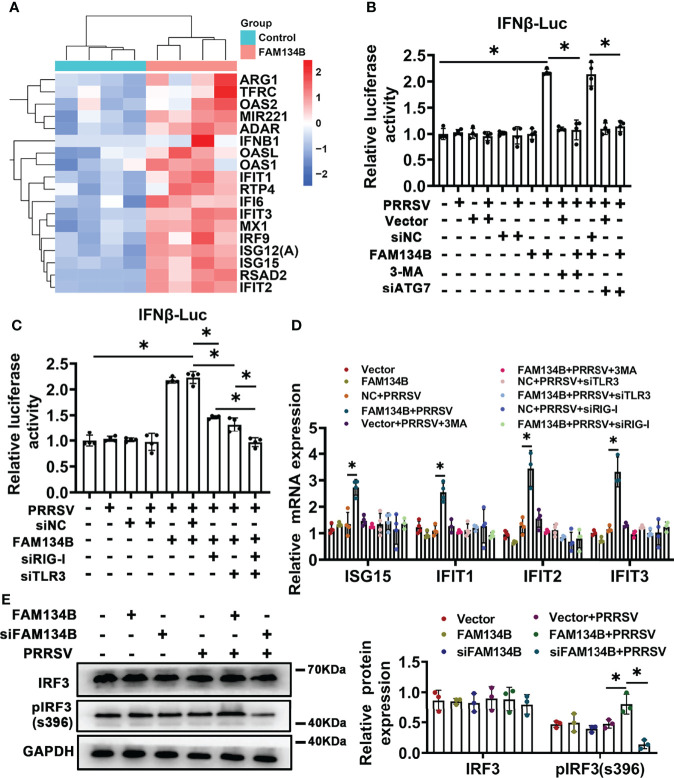
FAM134B activates type I interferon pathway through ER-phagy. **(A)** Heatmap of differentially expressed genes (DEGs) by RNA-seq. **(B, C)** Promoter luciferase activity of *IFNβ* by double luciferase reporting system. *P < 0.05, Wilcox test, n=4. **(D)** mRNA expression of *ISG15, IFIT1, IFIT2* and *IFIT3* in PK15^CD163^ cells. *P < 0.05, Kruskal Wallis test, n=3 or 4. **(E)** Expression of IRF3 and pIRF3(s396) in PK15^CD163^ cells. *P < 0.05, Kruskal Wallis test, n=3. GAPDH served as the control.

To reveal the molecular mechanism by which FAM134B as a host cell restriction factor inhibited PPRSV replication, we conducted transcriptomics analysis of PK15^CD163^ cells transfected with pcDNA3.1-FAM134B or control vector and infected with PRRSV (**Figure S4A**). A total of 230 differentially expressed genes (DEGs) were identified, of which 115 DEGs were upregulated and 105 were downregulated (Fold change >1.5 and *p*<0.05) (**Figure S4B**). Among 115 upregulated DEGs, the expressions of numerous interferon-stimulated genes (ISGs) such as *IFIT1*, *IFIT2*, *IFIT3*, *ISG15*, *ISG12(A)*, *OAS1, OAS2*, and *MX1* were significantly upregulated (**Figure 6A**). Furthermore, GO and KEGG pathway analyses showed that most DEGs were enriched in antiviral- and immune-related pathways (**Figure S4C**), suggesting that the overexpression of FAM134B activated type I interferon signaling pathway to inhibit PRRSV replication. Dual luciferase assay confirmed that overexpression of FAM134B in PRRSV-infected PK15^CD163^ cells activated IFNβ luciferase activity (**Figures 6B, C**). Consistently, quantitative PCR confirmed that the expressions of *ISG15, IFIT1, IFIT2*, and *IFIT3* were significantly increased in PRRSV-infected PK15^CD163^ cells transfected with FAM134B-overexpressed vector, and that the expressions of these ISGs were inhibited after the treatment with the autophagy inhibitor 3-methyladenine (3-MA) (**Figure 6D**). In addition, the treatments with 3-MA or siRNA targeting autophagy-related 7 (*ATG7*) inhibited the expressions of type I interferon signaling in PRRSV-infected PK15^CD163^ cells transfected with FAM134B-overexpressed vector (**Figure 6B** and **Figure S4D**), indicating that autophagy-related processes could regulate type I interferon production. The phosphorylation of IFN regulatory factor 3 (IRF3) plays a critical role in activating type I interferon signaling pathway. In this study, we found that the overexpression of FAM134B activated IRF3 phosphorylation in PRRSV-infected PK15^CD163^ cells, whereas siRNA-mediated depletion of FAM134B inhibited its phosphorylation (**Figure 6E**). Taken together, these results suggested that FAM134B could inhibit PRRSV replication by enhancing type I interferon signaling pathway. Autophagy mechanism regulates downstream TLR signaling and innate immune response (41). Further, we investigated whether FAM134B-mediated ER-phagy regulated host innate immune response by activating Toll-like 3 receptor (TLR3) and retinoic acid-inducible gene I (RIG-I) signaling pathways. The results showed that upon PRRSV infection, FAM134B could not activate host immune response after silencing expression of TLR3 or RIG-I (**Figures 6C, D** and **Figure S4D**). In addition, the expression level of IFNβ in the group with both TLR3 and RIG-I silenced was significantly lower than that in the group with TLR3 or RIG-I silenced alone (**Figure 6C**). Overall, these finding suggested that FAM134B-mediated ER-phagy could promote PRRSV recognition by TLR3 and RIG-I to activate type I interferon signaling, eventually inhibiting PRRSV replication.

## Discussion

ER is a continuous membrane-bound organelle, and it has multiple functions such as regulating RNA virus replication. RNA viruses can use ER to maintain a favorable environment for their survival and replication (42). In this study, we found that PRRSV infection subverted ER-phagy by attenuating expression of ER-phagy receptor FAM134B to promote virus replication. The downregulation of FAM134B expression caused expansion of ER, which might explain PRRSV infection-induced ER morphological change. Interestingly, RNA viruses can manipulate ER-phagy process by directly interacting ER-phagy receptors. For example, ZIKV, DENV, and WNV subvert ER-phagy through virally encoded proteases (14). Similarly, NS4B protein encoded by HCV interacts with ER-phagy receptor RTN3, which significantly affects viral replication (43). The control of ER-phagy receptor gene expression is also important for the regulation of ER-phagy, and FAM134B can be transcriptionally regulated by MiT/TFE factors to induce ER-phagy (44). TH domain-containing family protein 2 (YTHDF2) can downregulate the expression of FAM134B by recognizing the m6A tag on its transcripts (45). Our data showed that PRRSV suppressed the expression of FAM134B protein through post-transcriptional regulation. The upregulated miR-142-5p directly targeted the 3’UTR of *FAM134B* gene to downregulate expression of FAM134B protein in PRRSV-infected cells. This result provided new evidence that miRNAs played critical roles in the PRRSV-host interaction networks.

Autophagy plays an important role in RNA virus replication. Both non-selective autophagy and selective autophagy have impact on PRRSV infection. PRRSV NSP3 and NSP5 activate non-selective autophagy and induce the formation of autophagosomes (46). PRRSV infection partially inhibited the fusion of intracellular lysosomes and autophagosomes, thus resulting in the accumulation of autophagosomes, eventually providing more sites for PRRSV replication (47). In addition, PRRSV infection activated mitophagy or selective mitochondrial autophagy to block proapoptotic signaling, thus facilitating viral replication (48). Our results indicated that PRRSV infection subverted ER-phagy to enhance its replication. Silencing FAM134B by siRNAs or miR-142-5p significantly increased PRRSV replication in the infected cells. In contrast, overexpression of FAM134B enhanced ER-phagy to inhibit PRRSV replication. ER-phagy can effectively control ER quality by removing stressed and damaged ER, thus posing an obstacle to PRRSV replication. Autophagy is essential for innate immunity, and it is involved in a variety of innate immune pathways (49). Autophagy can effectively activate or inhibit IFN production by regulating the phosphorylation of IRF3 (50). Our results revealed the dual role of FAM134B in immune activation and autophagy. We observed that upon PRRSV infection, FAM134B effectively activated the expression of ISGs and the phosphorylation of IRF3, thereby promoting IFNβ expression, ultimately suppressing PRRSV replication. ER-phagy is considered as a special innate immune response, and it can directly digest ER subdomains and eliminate virus replication sites. Loss of FAM134B significantly increases viral load of EBOV and flaviviruses in the infected cells (29). Furthermore, PRRSV causes immunosuppression by interfering with IFN signaling pathways (9). Induction of type I IFN signaling pathway can significantly restrict PRRSV replication *in vitro* or *in vivo* (51, 52). One previous study has shown that upon viral infection, the activation of TLRs increases the production of IFN by inducing autophagy, and the negative regulation of autophagy can terminate or inhibit TLR signaling (53). Therefore, the recognition of pathogen-associated molecular patterns (PAMPs) is important for the initiation of antiviral innate immune response to PRRSV. TLRs and retinoic acid inducible gene I (RIG-I)-like receptors (RLRs), as the two most important classes of PRRs, play critical recognition roles in RNA virus infection. TLR3 located on the endosomal membrane can recognize dsRNA with a CpG motif and activate IRF3 to induce IFN production (54, 55). RIG-I, located in the cytoplasm, acts as a core sensor in response to RNA virus infection and activates IRF3 by binding to mitochondrial antiviral signaling protein (MAVS, located on the mitochondrial membrane), thereby inducing IFN production (56, 57). RNA pseudoknot region of PRRSV genome has been reported to be recognized as PAMP by TLR3 and RIG-I to activate type I IFN signaling pathway and induce ISGs (33), which was consistent with our findings that interference with RIG-I and/or TLR3 effectively inhibited the FAM134B-induced IFNβ expression. The ER autophagic mechanism can deliver viral PAMPs to PRRs to activate host immune response, thus restricting virus replication (33). Based on these findings, it could be concluded that FAM134B overexpression-induced ER-phagy might deliver PAMP of PRRSV to TLR3 and RIG-I, thus activating type I IFN signaling, eventually inhibiting PRRSV replication.

**Figure 7:**
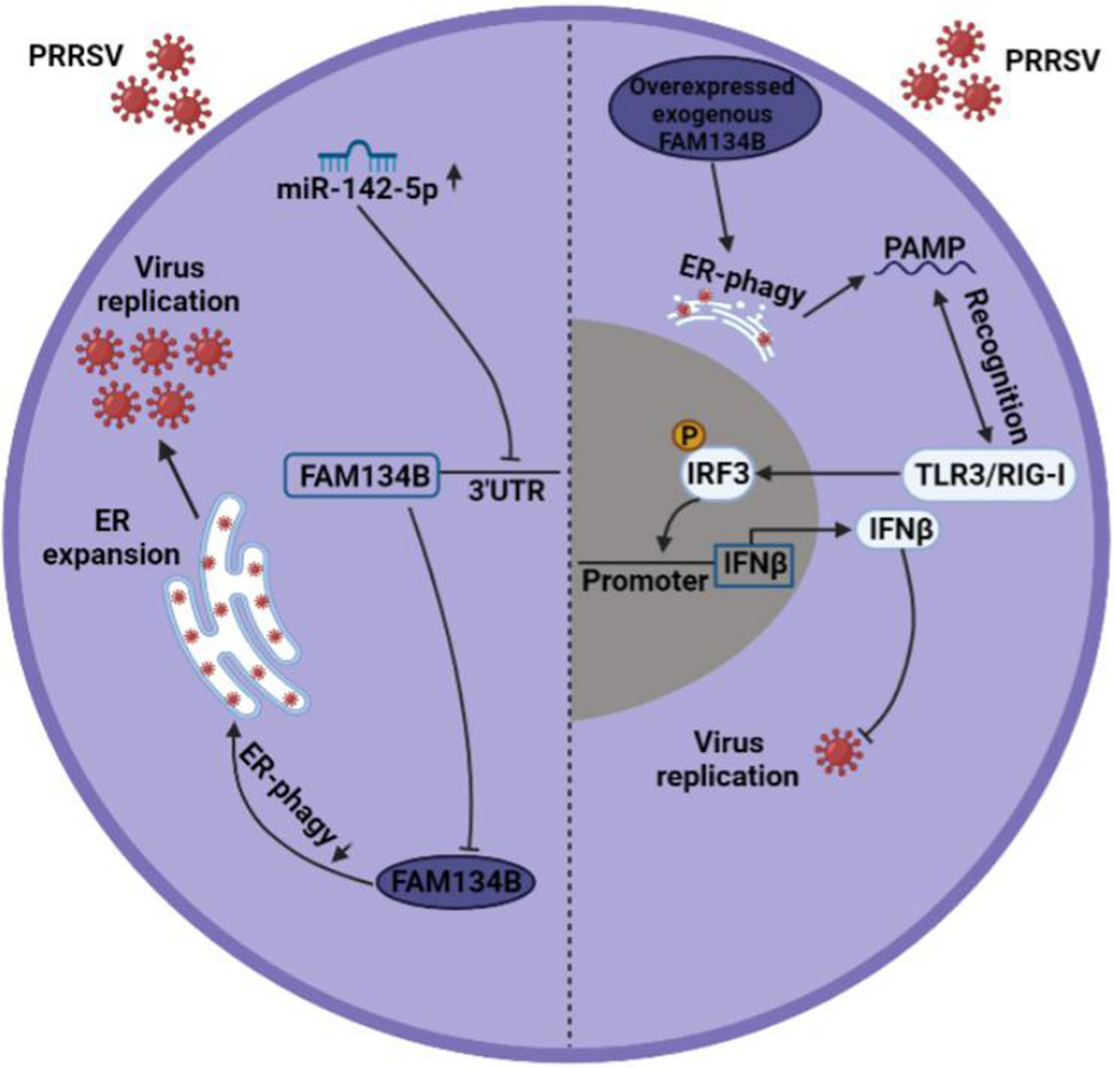
Mechanism underlying PRRSV pathogenesis regulation by FAM134B. In normal setting, PRRSV infection downregulated the expression of FAM134B (dark blue) *via* under the action of miR-142-5p, thereby initiating ER expansion and promoting PRRSV replication. In the scenario of FAM134B overexpression, PRRSV might be digested by FAM134B-induced ER-phagy, thereby generating the PAMP, which was recognized by TLR3/RIG-I to activate innate immune response and inhibit PRRSV replication.

In conclusion, our study revealed that PRRSV infection inhibited the expression of FAM134B by promoting miR-142-5p, thereby suppressing ER-phagy, ultimately facilitating PRRSV replication. Overexpression of FAM134B in PRRSV-infected cells could activate type I IFN signaling, ultimately inhibiting PRRSV replication (**Figure 7**). This study suggested that blocking virus-induced FAM134B downregulation and ER-phagy inhibition could be an effective strategy to suppress PRRSV replication. Our findings provide a theoretic basis for the development of PRRSV therapeutic targets.

## Data Availability Statement

The datasets presented in this study can be found in online repositories. The names of the repository/repositories and accession number(s) can be found below: https://www.ncbi.nlm.nih.gov/, NO. PRJNA773967 .

## Ethics Statement

The animal study was reviewed and approved by Institutional Animal Care and Use Committee of Huazhong Agricultural University.

## Author Contributions

XZ and BL designed and supervised the study; KG, QS, KK, XM, and XZ conducted experiments and interpreted the data; KG and XZ wrote the manuscript. All authors contributed to the article and approved the submitted version.

## Funding

This study was supported by the National Natural Science Foundation of China (32172699, 31930104 and 31802040) and the Major Project of Hubei Hongshan Laboratory (2021hszd019).

## Conflict of Interest

The authors declare that the research was conducted in the absence of any commercial or financial relationships that could be construed as a potential conflict of interest.

## Publisher’s Note

All claims expressed in this article are solely those of the authors and do not necessarily represent those of their affiliated organizations, or those of the publisher, the editors and the reviewers. Any product that may be evaluated in this article, or claim that may be made by its manufacturer, is not guaranteed or endorsed by the publisher.

## Supplementary Material

Supplementary Figure 1 PRRSV infection limits lysosomal degradation. Immunoblotting analysis of LC3-I and LC3-II proteins from PK15^CD163^ cells in different groups. **P* < 0.05, Kruskal Wallis test, n=3. GAPDH served as the control.

Supplementary Figure 2 Porcine FAM134B participates in ER-phagy. **(A)** Representative confocal images of PAMs, PK15, and MARC-145 cells at 48 h after transfection with eGFP-FAM134B (green) and mCherry-SEC61B (red) or endogenous CKAP4 (red). Scale bars: 20 μm. **(B)** Immunoblotting analysis of FAM134B, CKAP4, RTN4 proteins from PK15 cells with or without EBSS treatment at 0 h, 2 h, 4 h, and 6 h. **P* < 0.05, Kruskal Wallis test, n=3. GAPDH served as the control.

Supplementary Figure 3 FAM134B restricts PRRSV replication *in vitro*. **(A)** qPCR analysis of expression of *FAM134B* from PK15 cells treated with or without siFAM134B 1, 2, or 3. **P* < 0.05, Kruskal Wallis test, n=3. **(B)** Expression of PRRSV *ORF7* mRNA in PK15^CD163^ cells after PRRSV infection. PK15^CD163^ cells transfected with FAM134B expression vector or *FAM134B* siRNA were infected with PRRSV (MOI=1) for 48 h. **P* < 0.05, Kruskal Wallis test, n=3 or 4. **(C)** RNA copy number of PRRSV in the supernatant of infected cells transfected with siRNA-*FAM134B* or NC by absolute quantification PCR analysis. **P* < 0.05, Wilcox test, n=3. **(D)** Representative images of PRRSV-infected PK15^CD163^ cells at 48 h after transfection with siFAM134B or NC. **(E)** Expression of PRRSV *ORF7* mRNA from PRRSV-infected PK15^CD163^ cells transfected with empty vector, FAM134B-LIR-MUT, or pcDNA3.1-FAM134B. **P* < 0.05, Kruskal Wallis test, n=4. **(F)** Representative images of PRRSV-infected PK15^CD163^ cells at 48 h after transfection with empty vector, eGFP-FAM134B-LIR-MUT, or eGFP-FAM134B. The experiments were conducted with three or four independent replicates.

Supplementary Figure 4 FAM134B activates innate immune responses. **(A)** Schematic representation and immunoblotting assay of samples. **(B)** Volcano plot of differentially expressed genes from RNA-seq. **(C)** GO enrichment analysis of differentially expressed genes from RNA-seq. **(D)** Real-time PCR analysis of mRNA in PK15 cells transfected with siTLR3, siRIG-I, or siATG7. **P* < 0.05, Kruskal Wallis test, n=4. GAPDH served as the control in immunoblotting assay.

Supplementary Table 1 Antibody information.

Supplementary Table 2 Primers used for qRT-PCR.

## Abbreviations

DEGs, differentially expressed genes; DENV, dengue virus; CCER, mCherry-Cleaved ER-phagy Reporter; CHOP, C/EBP-homologous protein; CKAP4, cytoskeleton-associated protein 4; EATR, ER autophagy tandem reporter; EBOV, Ebola virus; ER, endoplasmic reticulum; ERAD, ER-associated degradation; FAM134B, Family with sequence similarity 134 member B; ISGs, interferon-stimulated genes; IRF3, IFN regulatory factor 3; IFNβ, interferon-beta; LC3, Microtubule-associated protein 1 light chain 3; LIR, LC3-interacting region; MOI, multiplicity of infection; PAMs, porcine alveolar macrophages; p-eIF2α, phosphorylated eIF2 alpha; p-IRF3, phosphorylated-interferon regulatory factor 3; PRRS, porcine reproductive and respiratory syndrome; PRRSV, porcine reproductive and respiratory syndrome virus; RIG-I, retinoic acid-inducible gene I; RHD, reticulon-homology domain; RTN4, reticulon 4; TLR3, Toll-like 3 receptors; UPR, unfold protein response; ZIKA, Zika virus; WNV, West Nile virus; 3-MA, 3-methyladenine.
