# Supplementary figures and images for: MiR-142-5p/FAM134B Axis Manipulates ER-Phagy to Control PRRSV Replication
Source: Front Immunol. 2022 Jun 20;13:842077. doi: 10.3389/fimmu.2022.842077 (PMC9251429; doi:10.3389/fimmu.2022.842077)

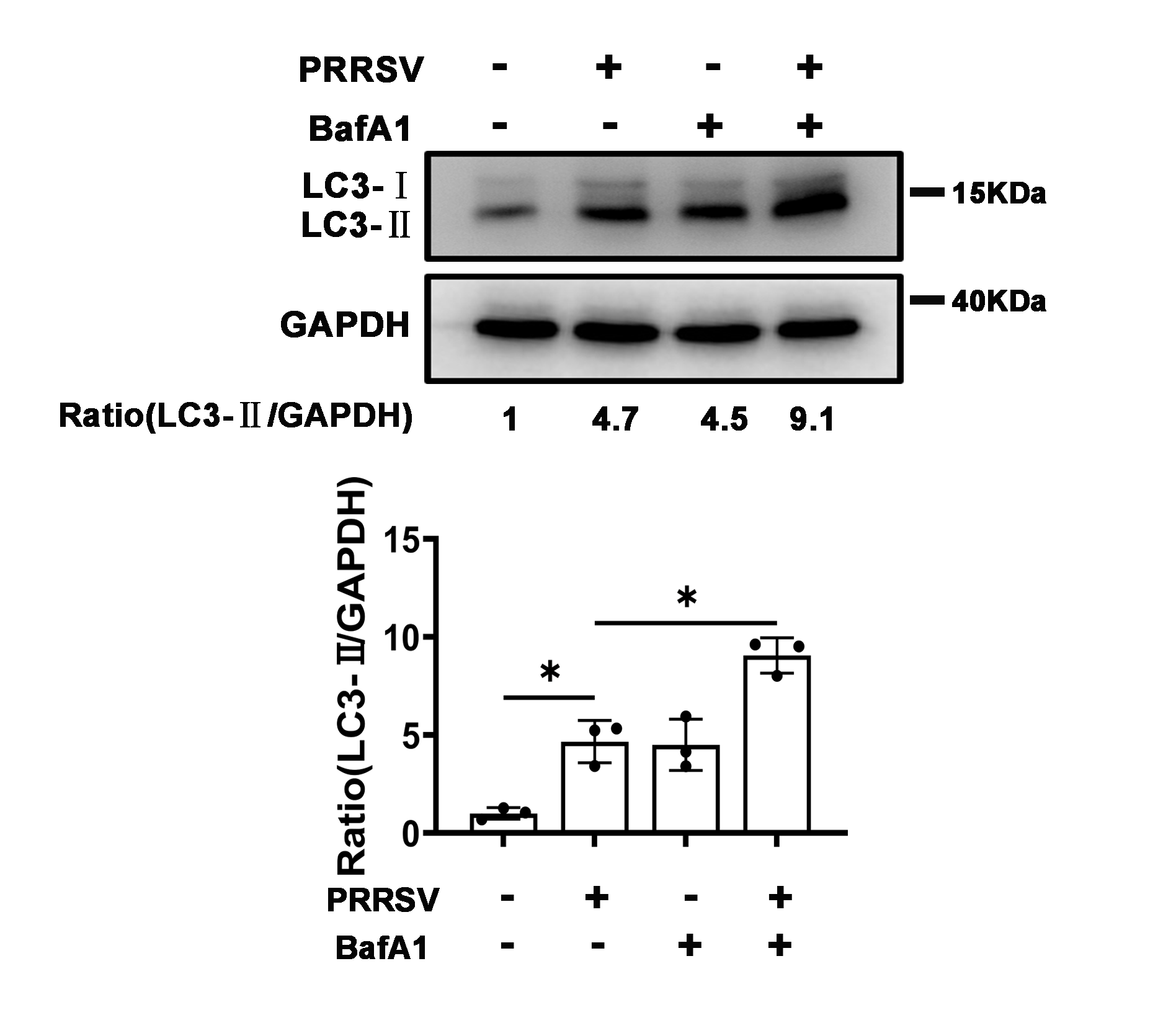

Supplement: Supplementary Figure 1 — PRRSV infection limits lysosomal degradation. Immunoblotting analysis of LC3-I and LC3-II proteins from PK15CD163 cells in different groups. *P < 0.05, Kruskal Wallis test, n=3. GAPDH served as the control. [file Image_1.tif]

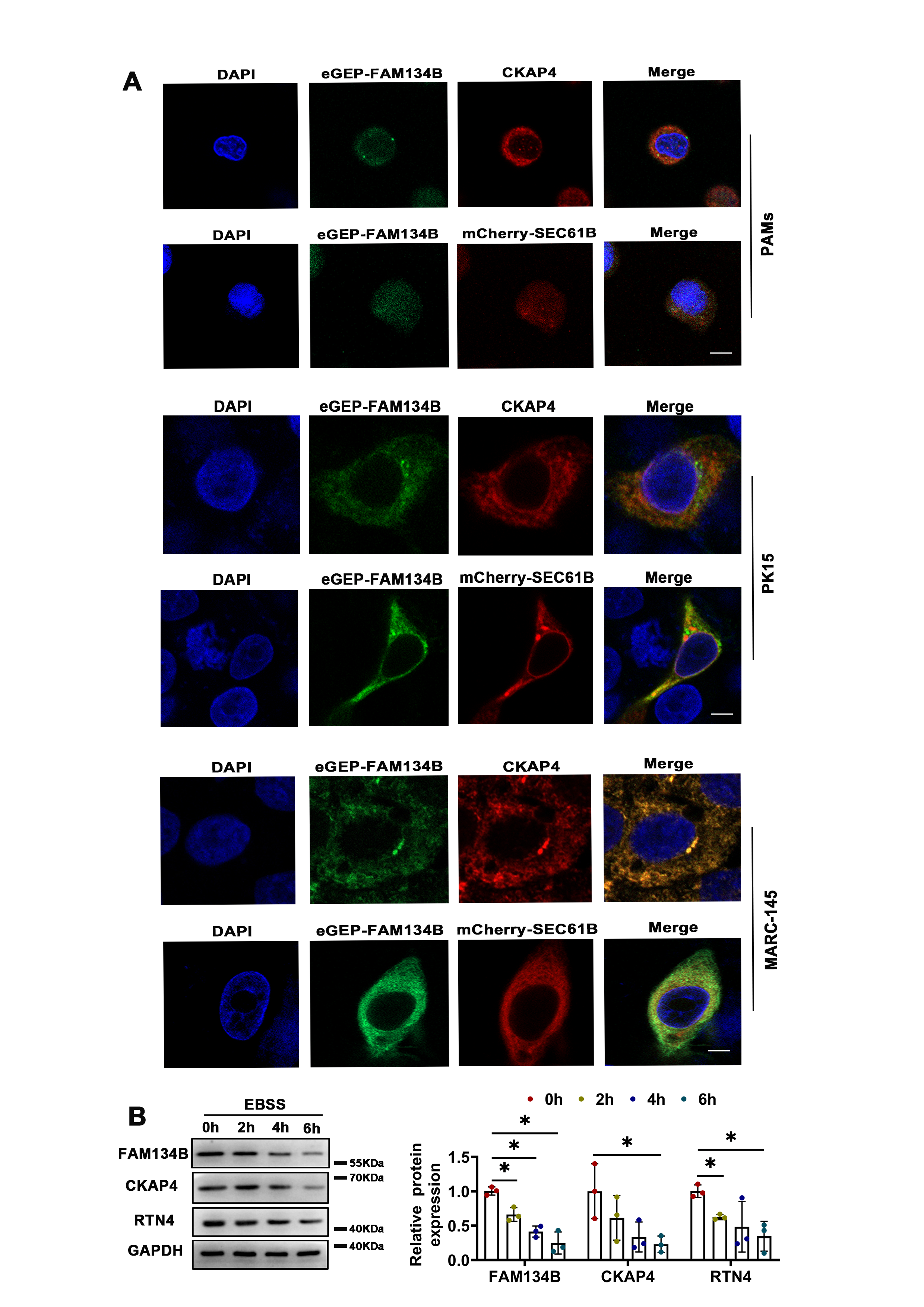

Supplement: Supplementary Figure 2 — Porcine FAM134B participates in ER-phagy. (A) Representative confocal images of PAMs, PK15, and MARC-145 cells at 48 h after transfection with eGFP-FAM134B (green) and mCherry-SEC61B (red) or endogenous CKAP4 (red). Scale bars: 20 μm. (B) Immunoblotting analysis of FAM134B, CKAP4, RTN4 proteins from PK15 cells with or without EBSS treatment at 0 h, 2 h, 4 h, and 6 h. *P < 0.05, Kruskal Wallis test, n=3. GAPDH served as the control. [file Image_2.tif]

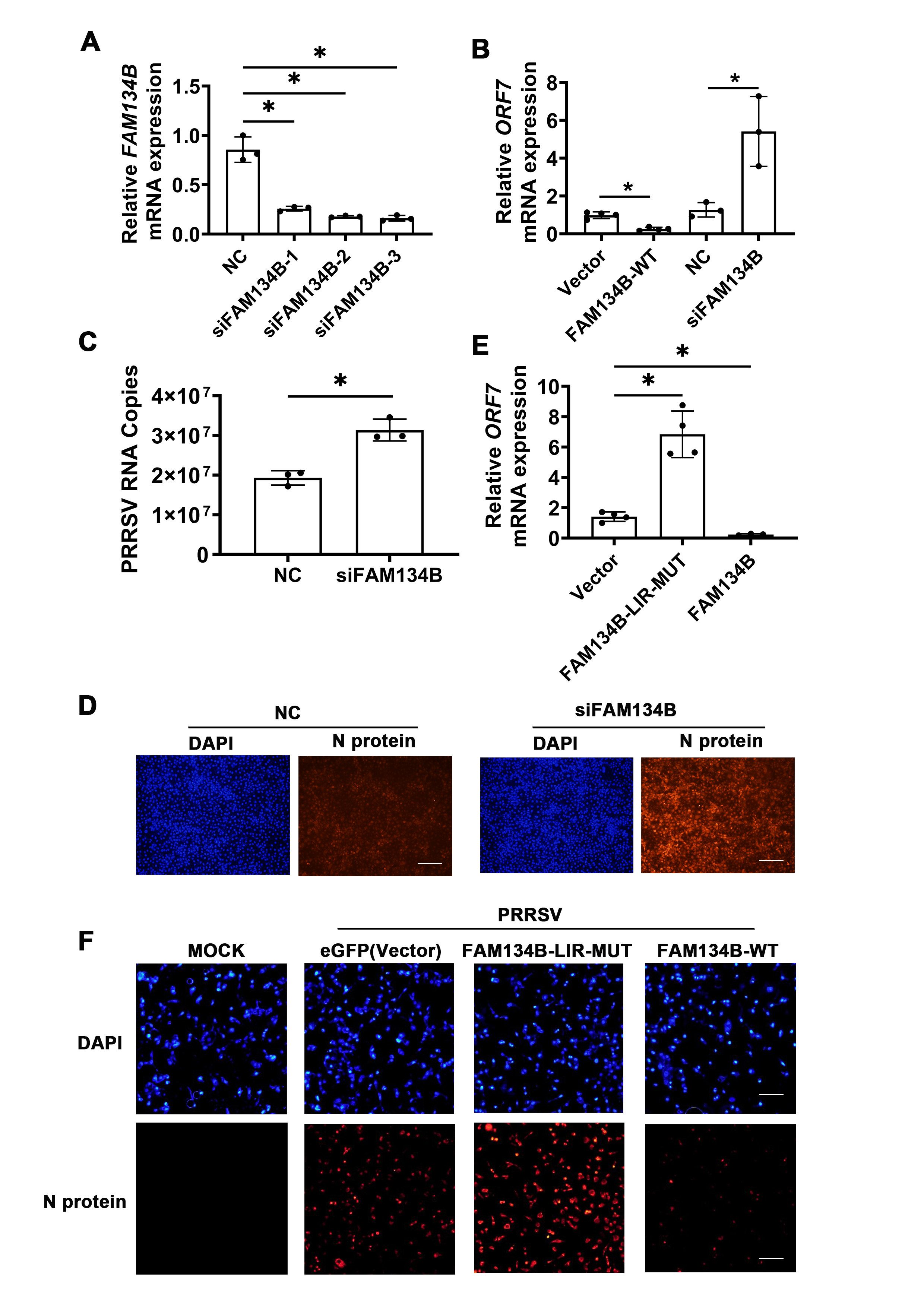

Supplement: Supplementary Figure 3 — FAM134B restricts PRRSV replication in vitro. (A) qPCR analysis of expression of FAM134B from PK15 cells treated with or without siFAM134B 1, 2, or 3. *P < 0.05, Kruskal Wallis test, n=3. (B) Expression of PRRSV ORF7 mRNA in PK15CD163 cells after PRRSV infection. PK15CD163 cells transfected with FAM134B expression vector or FAM134B siRNA were infected with PRRSV (MOI=1) for 48 h. *P < 0.05, Kruskal Wallis test, n=3 or 4. (C) RNA copy number of PRRSV in the supernatant of infected cells transfected with siRNA-FAM134B or NC by absolute quantification PCR analysis. *P < 0.05, Wilcox test, n=3. (D) Representative images of PRRSV-infected PK15CD163 cells at 48 h after transfection with siFAM134B or NC. (E) Expression of PRRSV ORF7 mRNA from PRRSV-infected PK15CD163 cells transfected with empty vector, FAM134B-LIR-MUT, or pcDNA3.1-FAM134B. *P < 0.05, Kruskal Wallis test, n=4. (F) Representative images of PRRSV-infected PK15CD163 cells at 48 h after transfection with empty vector, eGFP-FAM134B-LIR-MUT, or eGFP-FAM134B. The experiments were conducted with three or four independent replicates. [file Image_3.tif]

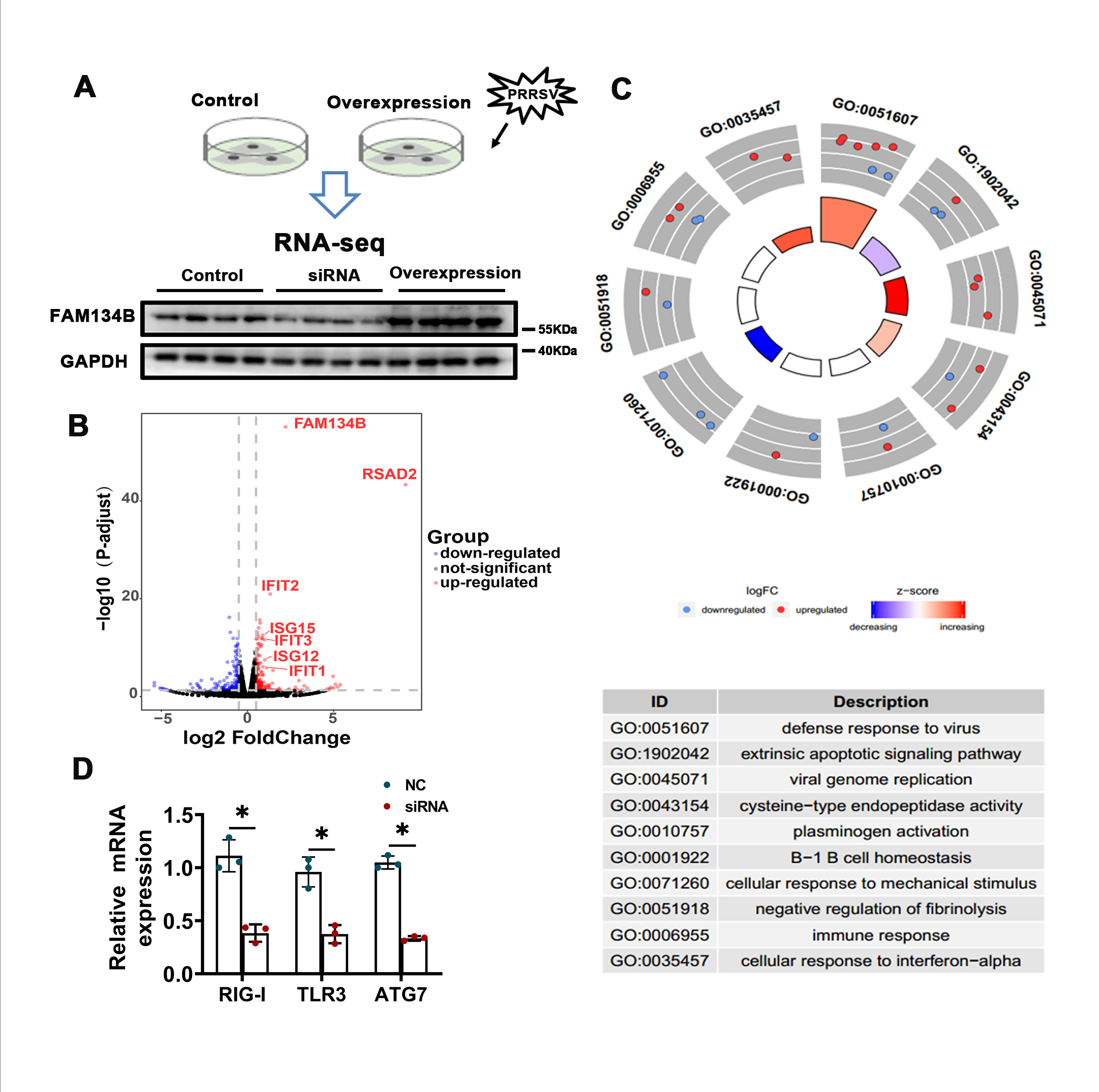

Supplement: Supplementary Figure 4 — FAM134B activates innate immune responses. (A) Schematic representation and immunoblotting assay of samples. (B) Volcano plot of differentially expressed genes from RNA-seq. (C) GO enrichment analysis of differentially expressed genes from RNA-seq. (D) Real-time PCR analysis of mRNA in PK15 cells transfected with siTLR3, siRIG-I, or siATG7. *P < 0.05, Kruskal Wallis test, n=4. GAPDH served as the control in immunoblotting assay. [file Image_4.tif]
