# Supplementary material for: MiR-142-5p/FAM134B Axis Manipulates ER-Phagy to Control PRRSV Replication
Source: Front Immunol. 2022 Jun 20;13:842077. doi: 10.3389/fimmu.2022.842077 (PMC9251429; doi:10.3389/fimmu.2022.842077)
Supplement: Supplementary Table 1 — Antibody information. [file Table_1.docx]

**Table S1. The information of antibody**

| **Antibody** | **Catalogue number**  **(Manufacture)** | **working dilution** |
| --- | --- | --- |
| FAM134B | 21537-1-AP(proteintech) | WB(1:1000);IF(1:100) |
| PRRSV Nucleocapsid protein | GTX129270(GeneTex) | WB(1:2000) |
| PRRSV Nucleocapsid protein | SDOW17(Rural Technologies) | IF(1:50) |
| CKAP4 | 16686-1-AP(proteintech) | WB(1:1000);IF(1:50) |
| RTN4 | 10950-1-AP(proteintech) | WB(1:1000);IF(1:50) |
| mCherry | ab183628(Abcam) | WB(1:1000) |
| LC3B | ab48394(Abcam) | WB(1:2000) |
| IRF3 | A0816(Abclonal) | WB(1:1000) |
| pIRF3(s396) | 29047(CST) | WB(1:1000) |
| GAPDH | 10494-1-AP(proteintech) | WB(1:5000) |
| HRP Goat Anti-Rabbit IgG (H+L) | AS014(Abclonal) | WB(1:3000) |
| FITC Goat Anti-Rabbit IgG (H+L) | AS011(Abclonal) | IF(1:100) |
| Cy3 Goat Anti-Mouse IgG (H+L) | AS008(Abclonal) | IF(1:100) |
| Cy3 Goat Anti-Rabbit IgG (H+L) | AS007(Abclonal) | IF(1:200) |
