# Supplementary material for: MiR-142-5p/FAM134B Axis Manipulates ER-Phagy to Control PRRSV Replication
Source: Front Immunol. 2022 Jun 20;13:842077. doi: 10.3389/fimmu.2022.842077 (PMC9251429; doi:10.3389/fimmu.2022.842077)
Supplement: Supplementary Table 2 — Primers used for qRT-PCR. [file Table_2.docx]

**Table S2. Primers used in qRT-PCR**

| **Primers** | **Primer sequence (5'-3')** |
| --- | --- |
| SUS-*GAPDH*-F | CGTCCCTGAGACACGATGGT |
| SUS-*GAPDH*-R  Homo-*GAPDH*-F  Homo-*GAPDH*-R | GCCTTGACTGTGCCGTGGAAC  AGAAGGCTGGGGCTCATTTG  AGGGGCCATCCACAGTCTTC |
| *ORF7*-F | CATCGCCCAACAAAACCAG |
| *ORF7*-R | CGTCGGCAAACTAAACTCCA |
| miR-142-5p-RT | CTCAACTGGTGTCGTGGAGTCGGCAATTCAGTTGAGAGTAGTGC |
| miR-142-5p–F | CTGGTAGGCATAAAGTAGAAAG |
| miR-142-5p-R | TCAACTGGTGTCGTGGAG |
| *U6*-94bp-F | CTCGCTTCGGCAGCACA |
| *U6*-94bp-R | AACGCTTCACGAATTTGCGT |
| *ISG15*-F | CGTGCAAGCTGACCAGTTCT |
| *ISG15*-R | CACGGTGCACATAGGCTTGA |
| *IFIT1*-F | TACATTTCCACTATGGCCGAT |
| *IFIT1*-R | GGCCTGCTCATAATACTCCA |
| *IFIT2*-F | AGGAACTAATAGGACACGCTCT |
| *IFIT2*-R | GATGGCCTTTTCTTCGCACT |
| *IFIT3*-F | GCAGCCAAATTTTACCGAGT |
| *IFIT3*-R | AGTCCTTGGCATATTTCCGTA |
| *RIG-I*-F  *RIG-I*-R  *TLR3*-F  *TLR3*-R | TATGTGCTCCTACTGGTTGTGGAAA  AGTTGAATAGCAAAAAAGACAACCT  CATTGAGAATCTATCCCTGAGCAA  TGAGGTTTGTCTGCTTTAGTCCAA |
